# Evolutionary conservation analysis between the essential and nonessential genes in bacterial genomes

**DOI:** 10.1038/srep13210

**Published:** 2015-08-14

**Authors:** Hao Luo, Feng Gao, Yan Lin

**Affiliations:** 1 Department of Physics, Tianjin University, Tianjin 300072, China; 2 Key Laboratory of Systems Bioengineering, (Ministry of Education), Tianjin University, Tianjin 300072, China; 3 SynBio Research Platform, CollaborativeInnovation Center of Chemical Science and Engineering, Tianjin 300072, China

## Abstract

Essential genes are thought to be critical for the survival of the organisms under certain circumstances, and the natural selection acting on essential genes is expected to be stricter than on nonessential ones. Up to now, essential genes have been identified in approximately thirty bacterial organisms by experimental methods. In this paper, we performed a comprehensive comparison between the essential and nonessential genes in the genomes of 23 bacterial species based on the Ka/Ks ratio, and found that essential genes are more evolutionarily conserved than nonessential genes in most of the bacteria examined. Furthermore, we also analyzed the conservation by functional clusters with the clusters of orthologous groups (COGs), and found that the essential genes in the functional categories of G (Carbohydrate transport and metabolism), H (Coenzyme transport and metabolism), I (Transcription), J (Translation, ribosomal structure and biogenesis), K (Lipid transport and metabolism) and L (Replication, recombination and repair) tend to be more evolutionarily conserved than the corresponding nonessential genes in bacteria. The results suggest that the essential genes in these subcategories are subject to stronger selective pressure than the nonessential genes, and therefore, provide more insights of the evolutionary conservation for the essential and nonessential genes in complex biological processes.

Essential genes are the genes that are indispensable for the maintenance of organisms. They play significant roles in many critical cellular processes, and hence are also considered the foundation of cellular life^1^. A wide variety of *in vivo* and *in vitro* approaches, including single-gene knock-out, transposon mutagenesis, antisense RNA and RNA interference, have been employed to identify the essential genes^2^. During the past decade, the application of the next-generation sequencing technology in the transposon mutagenesis has also facilitated various methods in the identification of the essential genes, such as TrsDIS, INSeq, HITS and In-seq^3^. As a consequence, the increase of the available essential genes promoted a broad spectrum of subsequent studies of essential genes, which are aiming at investigating the characteristics of the essential and nonessential genes. For instance, the essential genes are preferentially situated at the leading strand as well as in the cytoplasm, and enriched in protein complexes and enzymes^4^,^5^,^6^,^7^. Therefore, these outcomes have led to a development of the predictive models to identify the essential genes^8^,^9^,^10^,^11^. Additionally, the knowledge about the essential genes helps us to determine the universal minimal set of genes to sustain life and develop novel antibiotics to treat pathogenic bacterial infections, which will support the advancement of the pharmaceutical industry as well as the synthetic biology^12^.

It is well known that the rates of evolution have significant variations among protein-coding genes. If a protein plays a significant role in the cellular life, it should be under rigorous functional or structural constraints in response to the strong purifying (negative) selective pressure^13^. And its direct manifestation is the restriction of amino acid changes. The key principle for the identification of the essential genes is that the function absence of normal genes results in lethality or infertility in some special conditions^2^. Given these results, it is likely that the essential genes have a greater level of purifying selection pressure during the natural evolution. Some previous studies have reported that the essentiality of proteins plays an important role in the rates of evolution. Koonin *et al.* performed a genome-wide evolutionary analysis in three bacterial species, including *Escherichia coli*, *Helicobacter pylori*, and *Neisseria meningitides*, and found that the essential genes are evolutionarily conserved than the nonessential genes^14^. However, due to the limitation of data size at the time, a subset of the essential genes in their work were putative assumed based on the functional characteristics, rather than confirmed by experiments. Guo *et al.* also analyzed 16 different biological features on the evolutionary rate, and found that function essentiality is one of main contributors to the protein evolutionary rate variation^15^.

We constructed a database of essential genes named DEG, which has collected and organized the records of both essential genes and essential non-coding elements by genome-wide gene essentiality screens, including bacteria, archaea and eukaryotes^3^,^16^,^17^. In this study, along with the availability of the essential genes identified by experiment from the DEG database, the previous finding, that the essential genes are evolutionarily conserved than the nonessential genes, was confirmed with 23 genomes of bacterial organisms. Furthermore, we examined evolutionary conservation based on the clusters of orthologous groups of proteins (COGs), and found that the essential genes in the COG functional categories G, H, I, J, K and L tend to have a lower rate of evolution compared with the corresponding nonessential genes. The results suggest the difference between the essential and nonessential genes in terms of evolutionary rates among various functional categories, and provide further insights into the evolutionary pressures acting on the essential and nonessential genes.

## Methods

### Ka/Ks estimation

**Figure 1:**
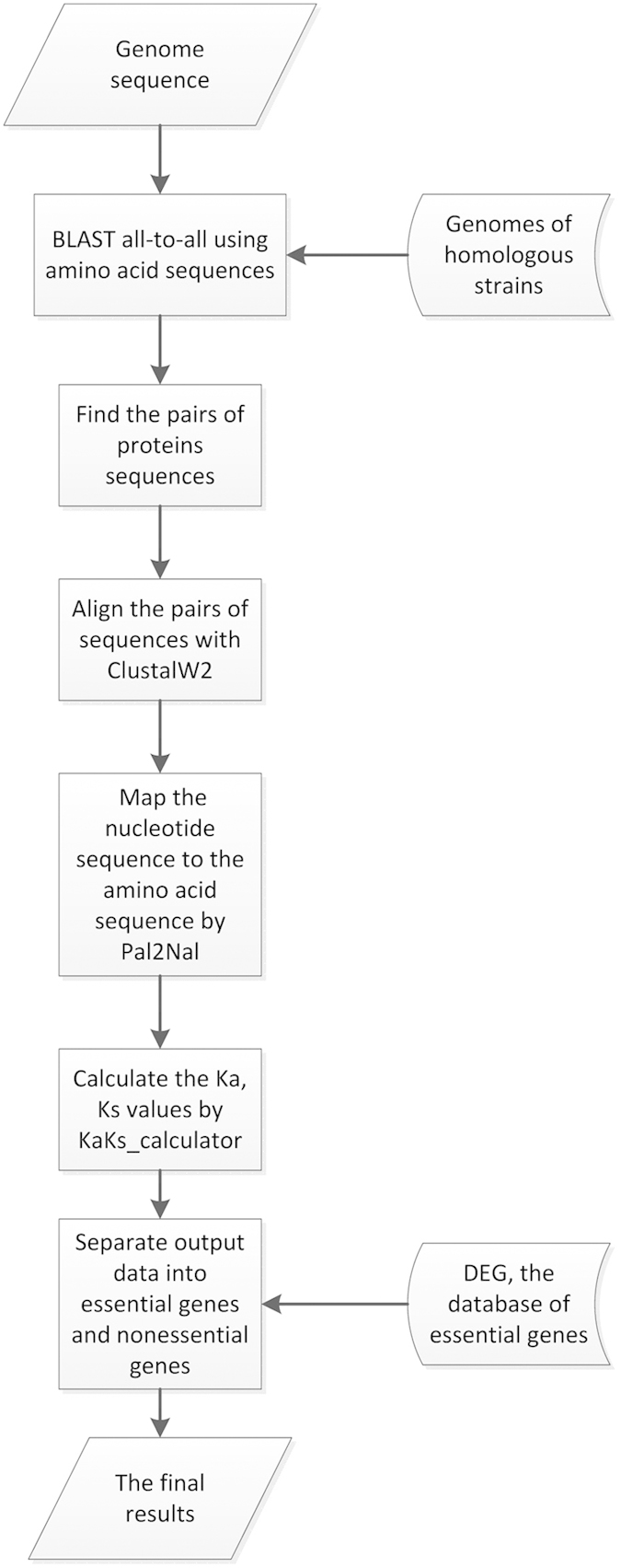
The workflow of the Ka/Ks estimation. Flow chart schematically shows the procedure to estimate the Ka/Ks ratios of all the protein-coding genes.

The Ka/Ks ratio is the ratio of the number of non-synonymous substitutions per non-synonymous site (Ka) to the number of synonymous substitutions per synonymous site (Ks), which could be used as an indicator of selective pressure acting on the protein-coding gene^18^. We developed a workflow to estimate the Ka/Ks ratio of all the genes in 23 bacterial organisms, whose essential and nonessential genes are available in DEG database. Figure 1 presents the procedure of the estimation. For each organism, we randomly picked at least one homologous strain to find pairs of orthologous proteins with E-value less than 10^−5^ based on BLASTP searches, and the orthologous protein with the highest score by BLASTP was selected for the further analysis^19^. All the complete genome sequences were downloaded via NCBI FTP from ftp://ftp.ncbi.nih.gov/genomes/Bacteria. The pairs of protein sequences were aligned by ClustalW2 with default options, and the nucleotide sequences were aligned to their corresponding amino acid sequences using Pal2Nal^20^,^21^. Ka/Ks value was calculated by KaKs_Calculator1.2 employing the Nei–Gojobori method^22^,^23^. And all the essential and nonessential genes were obtained from the latest release of DEG database, which is available at http://tubic.tju.edu.cn/deg/. During the Ka/Ks estimation, the majority of protein sequences in some homologous strains are precisely the same with the protein sequences in the original organism, so that the ratios of Ka and Ks of these homologous sequences could not be determined based on the Nei–Gojobori method. As a result, we only selected the strains that keep enough diversity in most protein-coding sequences.

### Bootstrap analysis

In order to estimate the difference of evolutionary conversation between the essential and nonessential genes in each organism, we performed a bootstrap analysis by half-sampling with replacement from the original gene set over 1000 replicates. The average values for these resampled sets including Ka, Ks and Ka/Ks were then calculated. It should be noted that the genes without valid Ka or Ks value were excluded from the analysis.

### COG analysis

The Clusters of Orthologous Groups of proteins (COGs) database is a useful tool for the functional annotation, and provides a consistent classification of bacterial and eukaryotic species based on orthologous groups^24^. In this study, all the essential and nonessential genes were divided into several functional subcategories based on the COG annotation (http://www.ncbi.nlm.nih.gov/COG/). It should be noted that owing to the absence of COG annotations, the genes of *Bacteroides fragilis* 638R were excluded in the COG analysis. The significance of difference for the Ka/Ks values between the essential gene and nonessential genes in each COG scope by organism was performed by Mann-Whitney U test. And the *P*-value less than 0.01 was considered statistically significant.

### Analysis tools

All the pipelines including BLASTP, ClustalW2, Pal2Nal and KaKs_calculator were executed by a custom python script. Biopython module was used to parse the GenBank and aln format files^25^. Statistical analyses were carried out using the Scipy and Pandas package. The figures were generated by the Python module Matplotlib^26^.

## Results and Discussion

### Essential genes are evolutionarily conserved than nonessential genes

**Figure 2:**
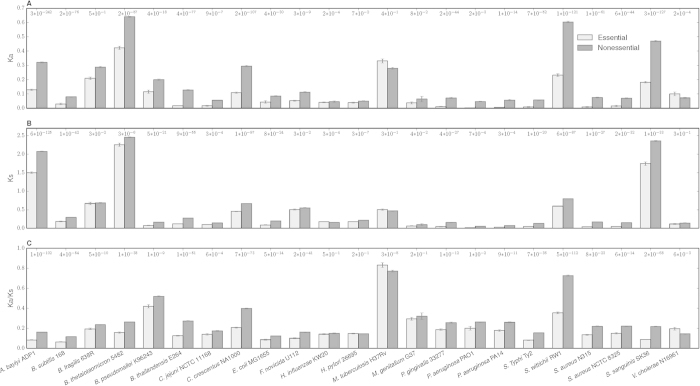
The average of Ka, Ks and Ka/Ks value for essential and nonessential genes in each organism. The histogram shows the averages for (**A**) Ka, (**B**) Ks and (**C**) Ka/Ks values between the essential and nonessential genes, respectively. The *P*-values calculated by Mann–Whitney U Test are also displayed at the top of figures.

In order to estimate the difference of evolutionary conservation between the essential and nonessential genes, we constructed a data set containing the essential and nonessential genes, which were determined in genome-wide screen. The gene set consists of more than 70,000 genes from 23 genomes of bacterial organisms. Table 1 represents the organisms used in this study. Then we identified and aligned more than 220,000 pairs of orthologous protein sequences based on BLASTP search, in which more than 180,000 pairs of proteins have valid synonymous (Ks) or nonsynonymous (Ka) substitution rates (see Methods). We also evaluated the properties of 180,000 pairs of proteins, and found that about 90% of them are with more than 30% amino acid identity and 50% minimum aligned residues by BLASTP searches. In addition, most of the rest of pairs with relative lower amino acid identity and length difference still have the same biological functions and COG assignments. This illustrates that with the condition of E-value less than 10^−5^, the most pairs of proteins we found are orthologous. Then the average Ka, Ks and Ka/Ks ratios of the essential and nonessential gene were calculated in each organism, and the levels of significance for the difference between the essential and nonessential genes were determined using the Mann-Whitney U test (Figure 2). Based on the results, we could find that the ratios of Ka and Ks show significantly lower level for the essential genes than for the nonessential genes in most organisms, which indicates that not only non-synonymous sites but also synonymous sites are subject to some degree of selection pressure. In addition, Student’s *t*-test was performed for the three averages between the essential and nonessential genes in all the organisms, and the differences are statistically significant (P = 0.0004, 0.001 and 0.0004, respectively). The lower three ratios of the essential genes, in particular, suggest that the essential genes are more conserved during the evolution, and consistent with the fact that the negative selection against amino acid replacements acting on the essential genes are more strict than on the nonessential genes.

**Figure 3:**
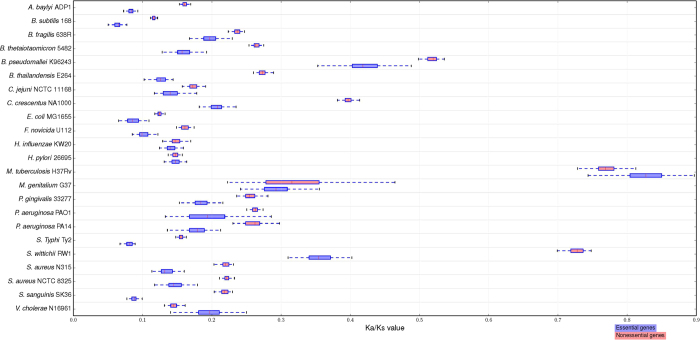
Box-plot diagrams for the differences between the essential and nonessential genes. The pairs of box-plots, presented in red and blue respectively, compare the distributions for the average Ka/Ks ratios of the essential genes and nonessential genes in each organisms.

In order to rule out the effect of the extreme values upon our results, the average values for Ka/Ks were evaluated by bootstrap analysis of 1000 replicates through half-sample resampling. Figure 3 reports the comparison for the distributions of the averages between the essential and nonessential genes by box plot. The result eliminates the influence of the abnormal values, and proofs that essential genes are often highly evolutionarily conserved than the nonessential genes across bacterial organisms to a certain extent.

However, in the Fig. 2, we also found a significant reduction in Ka, Ks and Ka/Ks for the nonessential genes than for the essential genes in *Mycobacterium tuberculosis* H37Rv and *Vibrio cholerae* N1696. The highly conserved nonessential genes, throughout distantly related bacteria, have been found in other organism, which are termed persistent nonessential (PNE) genes^27^. Due to the restrictions of current experimental techniques to define gene essentiality, the PNE genes are only dispensable for short-term survival and growth under laboratory conditions. Nevertheless, from an evolutionary point of view, PNE genes are also essential for successful survival of the population under various external environment^28^. With the aim of testing whether the abnormal results is related to the PNE genes, we performed a rough estimate of the proportion of the PNE genes in these organisms. In this study, the nonessential genes, which did not exhibit any variations with orthologous proteins in the nucleotide sequences (Ka, Ks = 0) and had homologous proteins in two or more organisms, were defined as PNE genes. Therefore, significantly higher percentages of the PNE genes for the two organisms than for other organisms are found, which means the PNE genes are enriched in the genomes of *M. tuberculosis* H37Rv and *V. cholerae* N1696 (See Table 1). And the enrichment of PNE genes indicates that a large amount of conserved nonessential genes were not recognized as experimentally essential genes, so the abnormal phenomenon do not conflict with the fact that the essential genes are evolutionarily conserved than the nonessential ones. In addition, for the pathogenic microorganisms, the most common antibiotics hit the only targets or pathways that are crucial for the organisms, and the proteins in these targets or pathways are often essential genes. As a result, the essential genes may be subject to the positive selection pressure, and evolve more rapidly than the nonessential genes in order to survive in some harsh and extreme environments. Finally, due to the limits of method, the selected homologous strains, which must keep enough diversity with original genome, may also have impact on the results. However, the conclusions drawn from the analysis of the evolutionary conservation in the 23 genomes of bacterial species are also valid.

### Evolutionary analysis of the essential genes based on the COG terms

**Figure 4:**
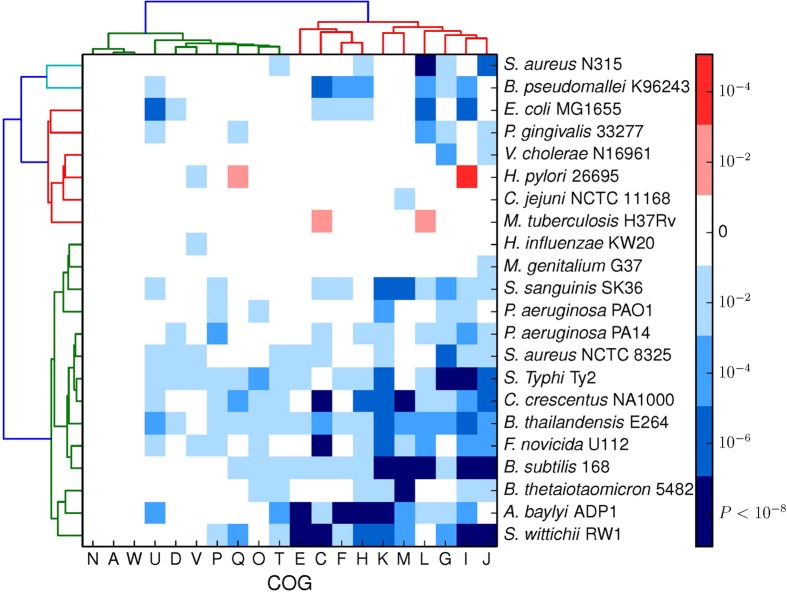
The heat map analysis for the significant conserved genes based on the COG categories. The hierarchal cluster diagram was constructed by Ward’s linkage clustering. The *P*-values of each COG category are calculated with Mann–Whitney U Test by organism, which reflect the significance of the difference for the Ka/Ks value between the essential and nonessential genes. The blue boxes represent that the COG subcategory in which the essential genes are evolutionarily conserved than the nonessential ones, while the red boxes represent the opposite case.

In order to further analyze the evolutionary conservation of the essential and nonessential genes in functional level, we classified all the essential and nonessential genes into 25 functional classes based on the COG subcategories. Consequently, a total of 54,175 genes in 22 organisms were classified in to 25 COG categories, while 11,359 genes had no COG assignments. Table 1 represents the numbers of COGs for the essential and nonessential genes in each organism. Then the Mann-Whitney U test was employed to test the significance of the differences between the essential and nonessential genes in each COG subcategory. The *P*-values less than 0.01 were considered statistically significant. A hierarchical clustering heat map, used to display the statistically significant COG categories in the 22 species of prokaryotic organisms, is present in the Figure 4. Note that the genes annotated by COG codes R and S were excluded in this study, because the two categories are denoted as unknown function and general prediction function. And the COG categories B, Y and Z were not considered due to the absence of available essential genes in them. In order to unify the standard of conserved function subcategories, the COG subcategories, in which essential genes are conserved than nonessential genes in more than half of all organisms, are defined as conserved function subcategories. The reason, that we did not used the clustering result to define the conserved subcategories, is that the different hierarchical clustering approaches would lead to different clusters. From the Figure 4, we could find that the essential genes from the functional subcategories with the COG codes G (Carbohydrate transport and metabolism), H (Coenzyme transport and metabolism), I (Transcription), J (Translation, ribosomal structure and biogenesis), K (Lipid transport and metabolism) and L (Replication, recombination and repair) are significantly conserved than the nonessential genes in more than half of all the organisms, and these COG subcategories are defined as conserved function subcategories in this research. Furthermore, the subcategories with COG codes C (Energy production and conversion) and U (Intracellular trafficking, secretion, and vesicular transport) also have statistical significance in no less than ten organisms. The COG subcategory M (Cell wall/membrane/envelope biogenesis) is conserved as well, because it is clustered into one group with other conserved subcategories based on the dendrogram and appears in nine organisms. Notably, the differences between the essential genes are not statistically significant in COG subcategories A (RNA processing and modification) and N (Cell motility). Due to only few genes annotated with COG code W (Extracellular structures), there is also no significant difference in this subcategory. In addition, the extraordinary circumstances, that the nonessential genes are conserved than the essential genes, are not extensively observed in this figure. It has been well known that the COG subcategories could be classified into four broad functional categories: (1) information processing and storage, (2) cellular processes, (3) metabolism, and (4) poorly characterized. The COG subcategories, in which the essential genes are significantly conserved than the nonessential ones, are mainly found in information processing and storage, and metabolism. Overall, from the results obtained so far, it seems that the highly evolutionary conservation for the essential genes than for the nonessential genes is often discovered in some central cellular mechanisms, which indicates that these biological processes expert stronger evolutionary pressure on the essential genes than on the nonessential genes.

## Conclusion

In the presented work, by comprehensively analyzing the Ka/Ks ratio of the essential genes in 23 species of prokaryotic organisms, we have demonstrated that the bacterial essential genes are evolutionarily conserved than the nonessential genes. Furthermore, the essential genes in COG subcategories of G, H, I, J, K and L present more evolutionary conservation than the nonessential genes, which indicates the essential genes are under stronger selection constraint in these biological processes. The results provide further insights into the evolutionary conservation of essential genes, and help to develop novel gene essentiality prediction algorithms.

## Additional Information

**How to cite this article**: Luo, H. *et al.* Evolutionary conservation analysis between the essential and nonessential genes in bacterial genomes. *Sci. Rep.* **5**, 13210; doi: 10.1038/srep13210 (2015).

## Figures and Tables

**Table 1:** The summary of the dataset^a^.

Organism	RefSeq	Essential		Nonessential		PNE^b^ (%)	RefSeq of the homologous strains^c^
		Genes	COGs	Genes	COGs		
*A. baylyi* ADP1	NC_005966	499	474	2594	1699	0.00	NC_009085 NC_011595 NC_017847
*B. subtilis* 168	NC_000964	271	267	3904	2483	0.15	NC_014479 NC_016047 NC_017195
*B. fragilis* 638R	NC_016776	547	–	3743	–	3.37	NC_003228 NC_006347 NC_009614
*B. thetaiotaomicron* 5482	NC_004663	325	256	4453	2578	0.00	NC_009614 NC_010831 NC_015164
*B. pseudomallei* K96243	NC_006351 NC_006350	505	453	5222	3724	21.52	NC_009078 NC_009076 NC_017831 NC_017831 NC_018527 NC_018529
*B. thailandensis* E264	NC_007651 NC_007650	406	372	5226	3706	0.00	NC_021173 NC_017832
*C. jejuni* NCTC 11168	NC_002163	228	178	1395	1000	0.86	NC_003912 NC_009707 NC_017280
*C. crescentus* NA1000	NC_011916	480	436	3224	1988	0.00	NC_002696 NC_010338 NC_014100
*E. coli* MG1655	NC_000913	296	284	4077	3144	1.84	NC_008253 NC_009801 NC_013008
*F. novicida* U112	NC_008601	392	340	1329	942	0.08	NC_017449 NC_017450 NC_017909
*H. influenzae* KW20	NC_000907	642	549	512	422	0.20	NC_007146 NC_017451 NC_022356
*H. pylori* 26695	NC_000915	323	230	1135	759	0.00	NC_017359 NC_019563 NC_022886
*M. tuberculosis* H37Rv	NC_000962	687	589	3070	1747	45.24	NC_017528 NC_021192 NC_021193
*M. genitalium* G37	NC_000908	381	287	94	64	39.36	NC_018495 NC_018496 NC_018497 NC_018498
*P. gingivalis* 33277	NC_010729	463	374	1627	840	1.23	NC_002950 NC_015571
*P. aeruginosa* PAO1	NC_002516	117	98	5454	4075	13.31	NC_017548 NC_018080 NC_021577
*P. aeruginosa* PA14	NC_008463	335	271	960	627	7.08	NC_002516 NC_017548 NC_018080
*S. Typhi* Ty2	NC_004631	358	338	3906	2707	3.84	NC_003197 NC_021151 NC_022544
*S. wittichii* RW1	NC_009511	535	452	4315	3184	–	NC_020561
*S. aureus* N315	NC_002745	302	280	2281	1471	13.42	NC_002951 NC_007795 NC_018608
*S. aureus* NCTC 8325	NC_007795	351	308	2541	1346	7.20	NC_002745 NC_018608 NC_020533
*S. sanguinis* SK36	NC_009009	218	211	2052	1341	0.00	NC_017595 NC_017618 NC_018526
*V. cholerae* N16961	NC_002506 NC_002505	779	433	2943	2173	47.54	NC_012578 NC_012580 NC_012582 NC_012583 NC_017269 NC_017270

^a^The organism name, RefSeq, proportion of PNE genes and the RefSeq used to evaluate conservation are provided. The count of the essential and nonessential genes as well as their COGs are also present in this table.

^b^The percentage is the proportion of PNE genes in the nonessential genes.

^c^The genome of *S. wittichii* RW1 only have single completely homologous genomes in NCBI, so that the percentage of PNE was not available. And due to the absence of homologous strains in *A. baylyi*, the three genomes were selected from the *Acinetobacter* genus.
